# Resistance of *M. leprae* to Quinolones: A Question of Relativity?

**DOI:** 10.1371/journal.pntd.0002559

**Published:** 2013-11-14

**Authors:** Nicolas Veziris, Aurélie Chauffour, Sylvie Escolano, Sarah Henquet, Masanori Matsuoka, Vincent Jarlier, Alexandra Aubry

**Affiliations:** 1 Université Pierre et Marie Curie-Paris 6, Paris, France; 2 Centre National de Référence de la Résistance des Mycobactéries aux Antituberculeux, Paris, France; 3 Groupe Hospitalier Pitié-Salpêtrière, Assistance Publique Hôpitaux de Paris, Laboratoire de bactériologie-hygiène, Paris, France; 4 Inserm, Centre for Research in Epidemiology and Population Health (CESP), U1018, Biostatistics, F-94807, Villejuif, France and Univ Paris-Sud, UMRS 1018, F-94807, Villejuif, France; 5 Leprosy Research Center, National Institute of Infectious Diseases, Higashimurayama-shi, Tokyo, Japan; Swiss Tropical and Public Health Institute, Switzerland

## Abstract

Multidrug resistant leprosy, defined as resistance to rifampin, dapsone and fluoroquinolones (FQ), has been described in *Mycobacterium leprae*. However, the *in vivo* impact of fluoroquinolone resistance, mainly mediated by mutations in DNA gyrase (GyrA_2_GyrB_2_), has not been precisely assessed. Our objective was to measure the impact of a DNA gyrase mutation whose implication in fluoroquinolone resistance has been previously demonstrated through biochemical studies, on the *in vivo* activity of 3 fluoroquinolones: ofloxacin, moxifloxacin and garenoxacin.

**Methodology/Principal Findings:**

We used the proportional bactericidal method. 210 four-week-old immunodeficient female Nude mice (NMRI-*Foxn1^nu^*/*Foxn1^nu^*) were inoculated in the left hind footpad with 0.03 ml of bacterial suspension containing 5×10^3^, 5×10^2^, 5×10^1^, and 5×10^0^ *M. leprae* AFB organisms of strain Hoshizuka-4 which is a multidrug resistant strain harboring a GyrA A91V substitution. An additional subgroup of 10 mice was inoculated with 5×10^−1^ bacilli in the untreated control group. The day after inoculation, subgroups of mice were treated with a single dose of ofloxacin, moxifloxacin, garenoxacin or clarithromycin at 150 mg/kg dosing. 12 months later mice were sacrificed and *M. leprae* bacilli were numbered in the footpad. The results from the untreated control group indicated that the infective inoculum contained 23% of viable *M. leprae*. The results from the moxifloxacin and garenoxacin groups indicated that a single dose of these drugs reduced the percentage of viable M. leprae by 90%, similarly to the reduction observed after a single dose of the positive control drug clarithromycin. Conversely, ofloxacin was less active than clarithromycin.

**Conclusion/Significance:**

DNA gyrase mutation is not always synonymous of lack of in vivo fluoroquinolone activity in *M. leprae*. As for *M. tuberculosis*, *in vivo* studies allow to measure residual antibiotic activity in case of target mutations in *M. leprae*.

## Author Summary

Although there is efficient multidrug therapy to cure leprosy, the transmission of *M. leprae* is still active, leading to 219,000 new cases in 2011. Drug resistant leprosy has been described and may prevent eradication of the disease, notably multidrug resistant defined as resistance to rifampin, dapsone and fluoroquinolones (FQ). Resistance to FQ is due to mutations in DNA gyrase. We used a mouse model to measure the impact of DNA gyrase mutations on *in vivo* FQ activity. All the FQ tested showed *in vivo* activity against the mutant tested (A91V mutant in subunit A of DNA gyrase). However, whereas ofloxacin was less active than the control treatment clarithromycin, it appeared that latter generation fluoroquinolones moxifloxacin and garenoxacin were as active as clarithromycin. Our results demonstrate that DNA gyrase mutation is not synonymous of total lack of *in vivo* FQ activity against *M. leprae*. Therefore, as for *M. tuberculosis*, *in vivo* studies are mandatory in order to measure the impact of DNA gyrase mutations on treatment efficacy against *M. leprae*.

## Introduction

*Mycobacterium leprae* is responsible for leprosy, that the World Health Assembly decided, in 1991, to “eliminate as a public health problem” by the year 2000. But, though a decreasing number of new cases registered each year (∼219,000) during the recent years [1], [2], it is generally admitted that the goal of leprosy elimination is far from being reached [3]. Future projections of the global leprosy burden indicates that 5 million new cases would arise between 2000 and 2020, and that in 2020 there would be still 1 million people with WHO grade 2 disability due to leprosy.

Reports from Asian countries with a high leprosy prevalence estimated rates of resistance at 15–20% to dapsone (DDS) and 3–8% to rifampin [4], [5]. Some studies estimated around 50% of resistance to DDS in relapsing cases [6], [7]. Although the exact magnitude of resistance to these drugs is difficult to assess, resistance in *M. leprae* is a concern particularly in relapsing multi-bacillary leprosy patients, by strongly reducing the possibilities of an effective treatment [1], [8]–[10].

Quinolones are good candidates for the development of more powerful treatments of leprosy, as demonstrated for moxifloxacin which is the only drug other than rifampin to be consistently bactericidal against *M. leprae* in clinical trials [11]. Fluoroquinolones play a crucial role in the treatment for drug-resistant leprosy and single-lesion new cases [11], but some quinolone-resistant *M. leprae* strains have been described [12], [13]. The mode of action of quinolones against *M. leprae* has been clearly identified and mechanisms of resistance have been investigated [4], [5], [12]–[17]. Substitutions within a highly conserved region of GyrA and possibly GyrB, which are subunits of the tetrameric DNA gyrase (A_2_B_2_), the so-called quinolone resistance-determining region (QRDR), are associated with the development of ofloxacin resistance in *M. leprae*, as demonstrated before in *M. tuberculosis*. Due to the lack of *M. leprae* growth in vitro, the exact impact of DNA gyrase mutations on fluoroquinolone susceptibility remains largely unknown. We previously demonstrated, using an enzymatic assay, that GyrA substitutions do not have the same impact on all the fluoroquinolones [15]. As an example, garenoxacin had the same inhibitory activity against *M. leprae* DNA gyrase carrying mutation implicated in resistance to ofloxacin as against wild-type enzyme, underscoring the potential advantage of this compound in leprosy [15].

The aim of the present study was to evaluate the bactericidal activities of several quinolones (i.e. ofloxacin, moxifloxacin and garenoxacin) against a *M. leprae* strain carrying an A91V GyrA substitution known to be involved in quinolone resistance [18]. Relation between GyrA A91V mutation and ofloxacin resistance has been extensively proven in the literature both in patients experiencing relapse after ofloxacin treatment (notably for strain for the strain used in the present work) [12], [18], [19] and through DNA gyrase inhibition in vitro [15]. We demonstrated that despite GyrA A91V mutation, garenoxacin and moxifloxacin maintained some *in vivo* activity.

## Materials and Methods

### Ethics statement

The laboratory has been approved on April, 24^th^ 2012 to carry out animal experiments. Nicolas Veziris who carried the animal experiments has the following license number: 75-1531. Aurélie Chauffour who performed the animal experiments has the following license number: B-75-1214. We followed the animal experiment guidelines of the Faculté de Médecine Pierre-et-Marie Curie. Animal experiments were performed in accordance with prevailing regulations regarding the care and use of laboratory animals by the European Commission. The experimental protocol was approved by the Departmental Direction of Veterinary services in Paris, France.

### Materials

Hoshizuka-4 strain is a multidrug resistant strain with mutation in the three main genes involved in *M. leprae* resistance to antibiotics: *folP* gene (P55S), involved in dapsone resistance; *rpoB* gene (S456L), involved in rifampicin resistance; and *gyrA* gene (A91V) involved in quinolones resistance (using numbering system of the *M. leprae* genome TN, GenBank nuNC002677). Briefly, Hoshizuka-4 strain was isolated from a patient who developed a lepromatous leprosy after repeated clinical relapses [18]. He received dapsone, streptomycin, rifampin, clofazimine, isoniazid, ofloxacin and prothionamide to treat subsequent relapses. These drugs were administrated irregularly as monotherapy or in combinations, often at doses below recommended levels and standard multidrug therapy was never applied. The drug resistant profile of the isolated strain was confirmed by the mouse footpad method (in nude mice) for fluoroquinolones (two fluoroquinolones, ofloxacin and sparfloxacin, were tested at 2 concentrations, 0.0001% and 0.001%) [18]. The GyrA A91V substitution corresponds to amino acid 90 and 83 in *M. tuberculosis* and *E. coli* numbering system, respectively.

Four week-old Nude mice (NMRI-*Foxn1^nu^*/*Foxn1^nu^*) were purchased from JANVIER breeding center, Le Genest Saint-Isle, France.

Ofloxacin was purchased from Sanofi-Aventis, France; moxifloxacin from Bayer Santé, France; garenoxacin from EasyBuyer LTD, China, and clarithromycin from Abbot France, France.

### Infection of mice with *M. leprae*

Animal experiments were performed in accordance with prevailing regulations regarding the care and use of laboratory animals by the European Commission. The experimental protocol was approved by the Departmental Direction of Veterinary services in Paris, France.

The ‘proportional bactericidal’ technique, described by Colston, allows measuring the bactericidal activity of a compound [20]. Mice are inoculated with serial 10-fold dilutions of the suspension of *M. leprae*. A group of mice were left untreated; the other mice are treated for a period of time that varies from a single dose to 60 days (usually 10 mice per dilution of inoculum for each treatment-group). After treatment, the mice are held for 12 months, to permit a single surviving organism to multiply to a readily detectable level (*M. leprae* divides every 14 days). Harvests of *M. leprae* are then performed from individual feet; the organisms are considered to have multiplied in those feet found to contain ≥10^5^ AFB. The proportion of viable *M. leprae* surviving treatment may then be calculated from the number of organisms that infects 50% of the mice. The proportion of viable *M. leprae* killed by the treatment is calculated by comparing the proportion of viable organisms in the treated mice to that in the control mice.

Two hundred and ten 4 week-old immunodeficient female Nude were divided among 5 groups, each containing four subgroups with 10 mice each. The mice of each subgroup were inoculated in the left hind footpad with 0.03 ml of bacterial suspension containing 5×10^3^, 5×10^2^, 5×10^1^, and 5×10^0^ *M. leprae* organisms of strain Hoshizuka-4 [18]. The suspension needed to inoculate mice was prepared from one footpad harvested from a nude mouse (according to the Shepard and Mac Rae method [21]), that had been inoculated one year earlier in the lab with 6.10^4^ AFB/footpad. Ten µl of the suspension were taken to create slides and AFB/footpad were counted after Ziehl-Neelsen coloration and ten-fold dilutions were made if it was necessary. All further ten-fold dilutions were made in Hank's balanced salt. A fifth subgroup of the untreated control group was inoculated with 5×10^−1^ Acid Fast Bacilli (AFB) per footpad.

### Treatment of mice

The day after inoculation, a table of randomization was created on website randomization.com in order to randomly allocate mice in different groups: untreated control, 10 mice per inoculum concentration from 5.10^3^ to 5.10^−1^ AFB (n = 50); treated mice, 10 mice for each inoculum ranging from 5.10^3^ to 5.10^0^ AFB (n = 40) and for each antibiotic: ofloxacin 150 mg/kg; moxifloxacin 150 mg/kg; garenoxacin 150 mg/kg; and clarithromycin 150 mg/kg included as a positive control. Single dose was given the day after inoculation and randomization, and all drugs were administrated by gavage in 0.2 mL sterile water as a single dose of antibiotic.

### Assessment of the effectiveness of treatment

Mice were held for 12 months, a period of time sufficient to permit multiplication of a single surviving organism to multiply to a readily countable level. At the end of this period, tissues from the footpad were removed aseptically and homogenized in a final 2 ml volume of Hank's solution as described by Shepard and McRae method [20]–[22]. *M. leprae* was considered to have multiplied (i.e., viable organisms survived the treatment) in those footpads found to contain ≥10^5^ bacilli.

### Molecular detection of second-step mutants

Total DNA was extracted from footpad of all mice inoculated with 5.10^3^ AFB, following the heat-shock procedure [23]. DNA was subjected to PCR amplifying the QRDRs in *gyrA* as previously described [10] and in *gyrB* using the following primers: GyrBlepS: 5′ ACG AGA GTT AGT GCG TCG AAA 3′ and GyrBlepAS: 5′ GCT GCG CTA AAA ACA CGT AC 3′. Typical reaction mixtures (50 µl) contained 0.5× reaction buffer, 2,5 mM of MgCl2, 0,25 mM of dNTPs, 0,4 µM of each primer (Eurofins MWG operon), 0,01 U of Taq polymerase (BIO X ACT SHORT TAQ POL, BIOLINE, France) and 5 ml of DNA extract. PCR-amplified fragments were purified by using QIAGEN DNA purification kit (QIAGEN, France) and sequenced by the dideoxy-chain termination method with the ABI PRISM BigDye Terminator v3.1 Cycle Sequencing Kit (Applied Biosystems, Courtaboeuf, France). The oligonucleotide primers used for DNA sequencing were those used for PCR. The nucleotide and deduced amino acid sequences were analyzed with the Seqscape v2.0 software (Applied Biosystems).

### Statistical analysis

The proportion of viable *M. leprae* organisms remaining after treatment was determined as the 50% infectious dose, and the significance of the differences between the groups was calculated by the method of Spearman and Kärber [22]. For multiple comparisons between the groups, Bonferroni's correction was applied, i.e., the difference would be significant at the 0.05 level only if the *P* value adjusted to the number of groups: 0.05/*n* in which *n* was defined as the number of primary comparisons. Thus the corrected *P* was 0.05/5 = 0.01.

## Results

### Mice survival

Fifty five mice died during the study. Twenty seven mice died due to their advanced age. Twenty eight died due to an accidental problem of water supply during the experiment: 4 mice in ofloxacin 5.10^3^ group, 7 mice in untreated control group 5.10^2^, 1 mouse in garenoxacin 5.10^1^ group, 1 mouse in garenoxacin 5.10^0^ group, 3 mice in clarithromycin 5.10^0^ group and 1 mouse in untreated control 5.10^−1^ group.

### Bactericidal activity

Results are presented in table 1. In the untreated control group there were 22.6% viable bacilli at the end of the 12 months. Clarithromycin killed 90% of viable bacilli, ofloxacin 73%, moxifloxacin 90% and garenoxacin 88%. Compared to untreated control group, the percentage of viable bacilli was significantly smaller in the following treated groups: p = 0.0005 for clarithromycin, p = 0.0005 for moxifloxacin, p = 0.0009 for garenoxacin. For ofloxacin the percentage of viable bacilli was smaller than that of control group but not after Bonferroni correction (p = 0.014). On the other hand, the percentage of viable bacilli was similar in the group treated by ofloxacin compared to groups treated by moxifloxacin and garenoxacin (p = 0.276 and 0.334).

**Table 1:** Bactericidal activities against *M. leprae* Hoshizuka-4 of ofloxacin, moxifloxacin and garenoxacin measured in mice by the proportional bactericidal method.

Treatment^a^	No. of footpads showing multiplication^b^ of *M. leprae*/no. of footpads harvested, by inoculum^f^					% Viable *M. leprae* c	p value^d^	% Viable *M. leprae* killed by treatment^e^
	5×10^3^	5×10^2^	5×10^1^	5×10^0^	5×10^−1^			
None (control)	6/6	3/3	9/9	5/7	0/9	22.60		
Clarithromycin	7/7	9/9	7/10	0/8		2.17	0.0005	90.39
Ofloxacin	1/1	8/8	9/9	1/7		6.06	0.014	73.18
Moxifloxacin	6/6	8/8	6/9	1/9		2.16	0.0005	90.44
Garenoxacin	6/6	10/10	6/9	1/8		2.7	0.0009	88.05

a All drugs were administered by gavage as a single dose.

b *M. leprae* bacilli were considered to have multiplied if the harvest from a footpad yielded ≥10^5^ acid-fast bacilli. When the maximum inoculum was 5×10^3^ bacilli per footpad, a proportion of viable *M. leprae* as small as 0.006% could be measured.

c Derived from the following equation: % viable *M. leprae* = 0.69/50% infectious dose [22].

d p value (comparison with control group).

e Calculated from the comparison of the proportions of viable organisms between untreated controls and the treated group.

f the high number of dead mice is due to an accidental problem of water supply that occurred during the experiment.

Clarithromycin was as active as garenoxacin (p = 0.723) and moxifloxacin (p = 0.757). Clarithromycin was more active than ofloxacin but not after Bonferroni correction (p = 0.034).

### Detection of second-step mutants

No mutation in *gyrA* or *gyrB* was found in mice footpads demonstrating the absence of second-step mutant selection in our experiment. This result is not surprising since a single pulse drug is unlikely to result in selection of second step mutations.

## Discussion

Phenotypic assessment of *M. leprae* drug resistance is usually done using the continuous method in the mouse footpad model [10], [18], [24]. This method does not allow assessing bactericidal activity. This study is the first, to the best of our knowledge, assessing *in vivo* the bactericidal activity of various antibiotics of the same family against a *M. leprae* strain carrying mutation involved in drug resistance. Although multiple doses of treatment have been used by others, we chose to treat mice with a single dose of fluoroquinolones in order to be able to compare our present results to our previously published results [25], [26]. We demonstrated that despite the presence of a GyrA substitution well known to confer FQ resistance, i.e. A91V [10], [13], [15], the 3 fluoroquinolones tested had still some in vivo activity (Table 1). Ofloxacin activity was marginally significant, but garenoxacin and moxifloxacin remained active. We don't believe that mortality due to the water supply problem biased significantly the ofloxacin results because, for all groups of mice, including the untreated control group, there was no difference between 5.10^2^ and 5.10^3^ inocula, and all of mice inoculated with 5.10^2^ and 5.10^1^ and receiving ofloxacin were positive. Therefore it's highly probable that all mice would have been positive in 5.10^3^ ofloxacin. Also the rank of activity between ofloxacin and moxifloxacin seen in wild-type strains in a previous study was maintained [27].

Despite the general rule of cross resistance between quinolones, garenoxacin, a new non-fluorinated quinolone, retains most of its activity against strains harboring QRDR mutations, in species such as *Streptococcus pneumoniae* and *Helicobacter pylori* [28]–[31]. This characteristic, combined with a lower rate of resistant mutant and favorable PK-PD parameters contribute to its higher activity against strains harboring DNA gyrase mutations compared to other quinolones. We previously demonstrated that garenoxacin has the same inhibitory activity against purified *M. leprae* DNA gyrase carrying mutation implicated in quinolone resistance than against the wild-type enzyme [15]. Although less active than moxifloxacin against wild-type strains [32], garenoxacin is as active as moxifloxacin against the GyrA A91V mutant. Garenoxacin is currently under development in several countries [33].

Surprisingly, moxifloxacin also retained most of its activity against the strain harboring the GyrA A91V substitution, despite this substitution reduces moxifloxacin inhibition activity against purified gyrase [15]. Two parameters could explain the maintained activity of moxifloxacin against the strain harbouring the GyrA A91V substitution. First, it should be kept in mind that moxifloxacin is more active than ofloxacin [25] and garenoxacin [32] against wild-type *M. leprae* [27]. More importantly, it is likely that moxifloxacin MIC against the mutant strain remains lower than moxifloxacin peak serum level, thus allowing some in vivo activity as already shown for *M. tuberculosis* [34].

Ofloxacin is naturally less active than moxifloxacin and garenoxacin against *M. leprae* [25], [27], [32]. In other bacterial species like *M. tuberculosis*, ofloxacin is also less active than mo, xifloxacin against susceptible strains and this difference remains also against strains harboring DNA gyrase mutations [35]. In other words, in case of DNA gyrase mutation, susceptibility decreases for both ofloxacin and moxifloxacin but the difference of activity between these 2 antibiotics remains the same and explains why despite maintained moxifloxacin activity, ofloxacin was only marginally active against *M. leprae*.

How these activities observed in a murine model can be translated in humans? We used Nude mice (NMRI-*Foxn1^nu^*/*Foxn1^nu^*) rather than Swiss or conventional BALB/c because this species is more sensitive for the detection of antibiotic activity [36]. As the strain used was multidrug resistant we could not use dapsone nor rifampin as controls and consequently chose clarithromycin. In a previous study conducted in Swiss mice inoculated with a wild-type *M. leprae* strain, the killing rate was lower for clarithromycin (75%) than for moxifloxacin (92%) [25]. In the present study, the killing rates were equivalent between these two antibiotics (Table 1). Thus GyrA A91V substitution reduced the activity of moxifloxacin *in vivo* that became equivalent to that of clarithromycin. In human, clarithromycin although less active than rifampin, has shown bactericidal activity [37]. Thus in case of multidrug resistance, moxifloxacin could still be used in combination with clarithromycin, in a second-line drug scheme against GyrA A91V mutants. We showed a similar phenomenon in *M. tuberculosis* in which the substitution A90V (equivalent to A91V in *M. leprae*) downgrades the bactericidal moxifloxacin into a bacteriostatic drug in immunocompetent Swiss mice [34].

An important point regarding translation of mouse results to human is the dosing of antibiotics used. The 150 mg/kg dosing used generates, in the mouse, an AUC (Area Under the Curve) equivalent to the 400 mg dosing in human for the 3 fluoroquinolones [34], [38]–[42]. For clarithromycin, the 150 mg/kg dosing generates an AUC equivalent to 500 mg twice-daily human dosing [43], [44]. Thus, for all tested drugs, the AUC, which is the main PK parameter predicting efficacy, was equivalent to AUC in human at the usual dosing of the antibiotic. Taken together these data indicate that the GyrA A91V substitution confers low-level fluoroquinolone resistance in *M. leprae* and that moxifloxacin can be used in humans against such mutant strains.

Molecular tools are more and more described and used for the diagnosis of drug resistance in leprosy [10], [13], [19], [45]. We demonstrated in the present study that detection of a mutation is not sufficient to exclude a drug from therapeutic regimen, especially when there are a few or no other alternatives. Regarding fluoroquinolones, the present study is relevant for leprosy since, in *M. leprae*, the GyrA A91V substitution is the most prominent substitution described in the literature [13], [19], [45], [46], while the other substitution found in *M. leprae* GyrA (G89C substitution, corresponding to G88C in the *M. tuberculosis* numbering system) has been described only once [13]. Based on results from enzymatic studies performed on *M. leprae* and *M. tuberculosis*, the latter substitution should decrease susceptibility to fluoroquinolones at least at the level obtained with GyrA A91V substitution and could lead to a high-level resistance phenotype [35]. Thus the drug-resistance level generated by DNA gyrase mutation probably differs depending on the mutations and the therapeutic consequences should also differ.

Equivalent in vivo experiments with *rpoB* and *folP* mutants would be important in order to measure the in vivo impact of drug resistance on rifampin and dapsone activity. Regarding rifampin, low-level resistance has been extensively studied in *M. tuberculosis*. For example, *M. tuberculosis* strains harboring *rpoB* L533P mutation display low-level rifampin resistance and the same mutation described in *M. leprae* [13] would deserve further evaluation.
